# Improving cellular uptake and bioavailability of periplocymarin-linoleic acid prodrug by combining PEGylated liposome

**DOI:** 10.1080/10717544.2022.2104406

**Published:** 2022-07-31

**Authors:** Huiyun Zhang, Shunru Wei, Yu Zhang, Anran Pan, Michael Adu-Frimpong, Congyong Sun, Gang Qi

**Affiliations:** a School of Chemistry and Chemical Engineering, Yancheng Institute of Technology, Yancheng, Jiangsu, China; b Department of Biochemistry and Forensic Sciences, School of Chemical and Biochemical Sciences, C. K. Tedam University of Technology and Applied Sciences (CKT-UTAS), Navrongo, Ghana; c Department of Central Laboratory, The Affiliated Huai’an No. 1 People’s Hospital, Nanjing Medical University, Huai’an, China

**Keywords:** Periplocymarin, small molecule prodrug, PEGylated liposome

## Abstract

Periplocymarin (PPM), a cardiac glycoside isolated from *Cortex periplocae*, has a strong anti-tumor effect against various cancer cells. However, cardiotoxicity and rapid metabolism hinder its clinical applications. In this study, small molecule prodrug was integrated into PEGylated liposome to improve the efficiency of periplocymarin *in vivo*. The periplocymarin-linoleic acid (PL) prodrug was constructed by conjugating the linoleic acid with PPM via esterification, which was further facilitated to form PEGylated liposome (PL-Lip) through film dispersion. Compared with PL self-assembling nano-prodrug (PL-SNP), PL-Lip showed better colloid stability, sustained drug release kinetics, and enhanced cellular uptake by tumor cells. Notably, PL-Lip performed better than PPM and PL-SNP in terms of tumor distribution and pharmacokinetics, which include bioavailability and half-life. Altogether, the prodrug PEGylated liposome represents a good strategy and method for long-circulating and tumor-targeting delivery of periplocymarin with enhanced clinical application prospect.

## 1. Introduction

Periplocymarin (PPM) which was isolated from *Cortex periplocae* showed a strong antitumor activity on various cancer cell. However, some problems were encountered, such as systemic toxicity and unsatisfactory pharmacokinetics, namely short circulating half-life and poor tumor distribution (Zhang et al., 2016). In the light of the problems above, nano preparation maybe a good solution due to its enhanced bioavailability, lower toxicity and enhanced permeability and retention (EPR) effect caused by its small size effect (Gu et al., 2021). But, the partially hydrophobic and rigid structure of PPM has been hardly loaded into the nano-preparation include liposome and nanoparticles (with drug loading efficiency less than 1%).

Small molecule prodrug refers to chemically modified, bioinert small molecule drugs which could improve the physicochemical property and physiological disposition of drug (Li et al., 2021). At present, the combination technology of small molecule prodrug and nano preparation has been applied in enhancing the security and tumor targeting of commonly used chemotherapy drugs paclitaxel and doxorubicin hydrochloride (Huang et al., 2014). Some prodrug such as periplocymarin-linoleic acid and periplocymarin-Vitamin E were developed to nanoprodrug in our previous research (Zhang et al., 2017, 2020). Periplocymarin-linoleic acid was synthesized by direct esterification. Its synthetic process is simple and suitable for industrial production. What is more, periplocymarin-linoleic acid can self-assemble into nanoparticles which have many advantages of both prodrug and nano preparation. In addition, prodrug can solve the above problem of difficult encapsulation for periplocymarin. However, nanoprodrug is unstable and easily cleared by the reticular phagocytic system, which may cause low utilization rate in the treatment of cancer.

In order to overcome the above drawbacks of nanoprodrug, many approaches such as lipid emulsion, PEGylated-nanoparticles and PEGylated liposome have been investigated in taxane-based prodrug such as docetaxel, paclitaxel, and cabazitaxel (Luo et al., 2016; Ren et al., 2016; Xie et al., 2020). Among these, liposomes have recently attracted great attention in the fields of drug delivery system (Wang et al., 2017). Compared to other nano-preparation, PEGylated liposomes can keep both the fusion ability with tumor cell of liposome and long cycle characteristic in blood of PEG modification (Large et al., 2021). Therefore, in this study, periplocymarin-linoleic acid loaded PEGylated liposome (PL-Lip) was designed to increase drug loading of PPM and stability, cellular uptake, *in vitro* and *in vivo* circulation time of PL. Thus, this study provides an important research basis for the ultimate application in tumor therapy.

## 2. Materials and methods

### 2.1. Materials

Periplocymarin-linoleic acid (PL) and PL-SNP were prepared according to our previous report (Zhang et al., 2020). DSPE-PEG_2k_ was obtained from A.V.T. Pharmaceutical Technology Co., Ltd (Shanghai, China). Linoleic acid, cholesterol, and phospholipid (soybean lecithin, 98%) were obtained from Macklin Industrial Co., Ltd (Shanghai, China).

### 2.2. Preparation of PL-Lip

PEGylated liposomes were prepared based on the previous method with some slight modification (Gao et al., 2021). In brief, PL (4 mg), phospholipid (20 mg, soybean lecithin, 98%) and DSPE-PEG_2k_ (2 mg) and cholesterol (10 mg) were dissolved in 10 mL of anhydrous alcohol by ultrasound. The solution was rotary-evaporated using a rotary evaporator to remove ethanol. At the bottom of the flask, a film was formed and vacuum dried for several hours. Double distilled water was added and hydrated for many minutes. Finally, the PEGylated liposome solution was filtered through a 0.45 μm membrane to obtain PL loaded PEGylated liposome (PL-Lip) solution.

### 2.3. The analysis of characterization and encapsulation efficiency

The hydrodynamic diameters and zeta potential of PL-Lip and PL-SNP were assessed using a Zeta Potential/Particle Size analyzer (Malvern Instruments, Malvern, UK). The morphological characteristics of PL-Lip and PL-SNP were observed under TEM using a JEOL model JEM-1400 Plus at an acceleration voltage of 100 kV. The sample was placed on a copper grid and dried under the lamp, then subjected to negative dyeing with phosphotungstic acid solution. For the estimation of encapsulation efficiency, PEGylated liposome solution (4 mg·mL^−1^) was filtered to remove the unentrapped drug. The drug concentration was measured with HPLC method described in Supplemental material. The encapsulation rate (EL, %) was calculated according to the following formula:

$$\text{EL}\left(\%\right)\;=\;\left(X_{\text{1}}/X_{\text{2}}\right)\;\times \text{ 1}00\%.$$

where *X*_1_ represented the weights of total prodrugs in PEGylated liposome after filtration, and *X*_2_ stood for the initial prodrug added for encapsulation.

### 2.4. The stability of PL-Lip and PL-SNP

The long-time colloidal stability of the prepared PL-Lip and PL-SNP were carried out for 30 days. Concisely, aliquot (1 mL) of PL-Lip or PL-SNP was added into 10 mL of water or PBS (pH = 7.4) and stored at 4 °C. The PL-Lip was incubated for 24 h at 37 °C with gentle shaking. At predetermined time points (0, 6, 12, and 24 h), the particle size was determined by Malvern Zeta Sizer.

### 2.5. *In vitro* release of PL-Lip and PL-SNP

The *in vitro* release test was carried out by dialysis. A total of 1.0 mL of sample solution was put into a dialysis bag (MV 3500 D, 25 mm × 5 m). The dialysis bags were immersed in 80 mL of PBS (pH = 7.4) containing 0.5% tween 80 with or without esterase. The test was performed on a water bath thermostatic vibrator at 37 °C with a constant speed of 100 rpm. At time points of 0.25, 0.5, 0.75, 1, 2, 4, 6, 8, 10, 12, 24, 36, and 48 h, 2 mL of the medium was removed and replaced with an equal volume of fresh medium. The samples were filtered and determined via the HPLC method stated in Supplemental material.

### 2.6. *In vitro* cytotoxicity

HepG2 cells were cultured in DMEM supplemented with 10% (v/v) FBS, penicillin (100 units/mL) and streptomycin (100 µg/ml) at 37 °C under a humid atmosphere containing 5% CO_2_. The *in vitro* cytotoxicity of the prepared formulations in HepG2 cells was measured with MTT. Briefly, cells were seeded into 96-well plates at a density of 4 × 10^3^ cells/well and incubated at 37 °C for 24 h. Later, the cells were treated with 100 µL medium containing each of serial dilutions of samples (free PPM, Blank Lips, PL-SNP, and PL-Lip). Afterwards, the cells were cultured for 48 or 72 h. Subsequently, 20 µL of MTT solution (5 mg/mL) was added to each well and incubated at 37 °C for additional 4 h. Then, DMSO (100 µL) was used to solubilize the formazan crystals, wherein the absorbance was determined at 595 nm using a microplate reader. The cell viability rate (VR) was calculated according to the following formula: cell viability (%) = *A*_sample_/*A*_control_×100%, where *A* refers to the absorbance value.

### 2.7. *In vitro* cellular uptake

The cellular uptakes of PL-SNP and PL-Lip were measured with a fluorescence microscope after encapsulating the curcumin 6 (C-6). HepG2 cells were seeded into 6-well plates at a density of 1 × 10^6^ cells/well. When the cells confluence reached 80%, the cells were washed and incubated with free C-6, C-6@PL-SNP and C-6@PL-Lip at an equivalent C-6 dose of 200 ng mL^−1^ for 3 h. Afterwards, the cells were washed for three times and fixed with 4% formaldehyde, and the nuclei were stained by Hoechst 33342. After being washed with PBS, the fluorescent in cells were examined with a confocal laser scanning microscopy (TCS SP5 II, Leica, Germany).

### 2.8. Pharmacokinetic analysis

Fifteen healthy male SD rats were acclimatized to the environment for three days according to the guidelines of the Jiangsu Council on Animal Care. The rats (220 ± 20 g, *n* = 5 for each group) were injected into the tail vein with PPM solution, PL-SNP and PL-Lip at a dose equivalent to 4 mg/kg of PPM, respectively. During the indicated time intervals (0.25, 0.5, 1, 2, 4, 6, 8, 10, and 24 h), blood samples (0.5 mL) were collected into heparin anticoagulant tube from the posterior plexus vein of rats, before 10 min of centrifugation at 3000 rpm to obtain the plasma. Sample pretreatment was proceeded as indicated in our previous report (Zhang et al., 2020). The drug concentration in blood was measured via the HPLC analysis method described in Supplemental material.

### 2.9. Tissue biodistribution study

The tumor model was developed as stated in our previous study (Zhang et al., 2020). We injected into the tail vein of H_22_-tumor bearing mice with free PPM, PL-SNP, and PL-Lip at a dose equivalent to 4 mg/kg of PPM, accordingly. At time-controlled periods (0.5, 1, 2, and 4 h) after administration, we sampled blood from mice, and then the tissues were taken after the mice have been sacrificed. All the tissues were cleaned with ice-cold PBS solution and store at −20 °C. The sample pretreatment and content determination measurement was described in our previous report (Zhang et al., 2017).

Free C-6 solution and C-6@PL-Lip were injected at a dose equivalent to 1 mg kg^−1^ of C-6. Tissue and blood were collected from mice after injection at 24 h point. Tissues were immediately weighed and cleaned with 0.9% NaCl solution. Afterwards, the tissues were homogenized in saline solution to obtain 0.2 g/mL tissues homogenate. The C-6 concentration was determined using the HPLC method (Wang et al., 2014).

## 3 Results and discussion

### 3.1. Preparation and characterization of PL-Lip

The PL-Lip was prepared through the film dispersion method according to the reported methods with some slight modification. The mass ratio of lecithin, cholesterol, DSPE-PEG2k, and PL was selected as 10:5:1:2 based on EE using a single factor experiment. DSPE-PEG2k was commonly used as part of the phospholipid membrane to prepare PEGylated modified liposome. Some reports indicated that DSPE-PEG2k modified liposome achieved the effect of “invisibility” *in vivo* because it prevents the plasma protein from absorbing liposome surface and avoids the rapid phagocytosis of the mononuclear macrophage system (Fang et al., 2016).

**Figure 1.**
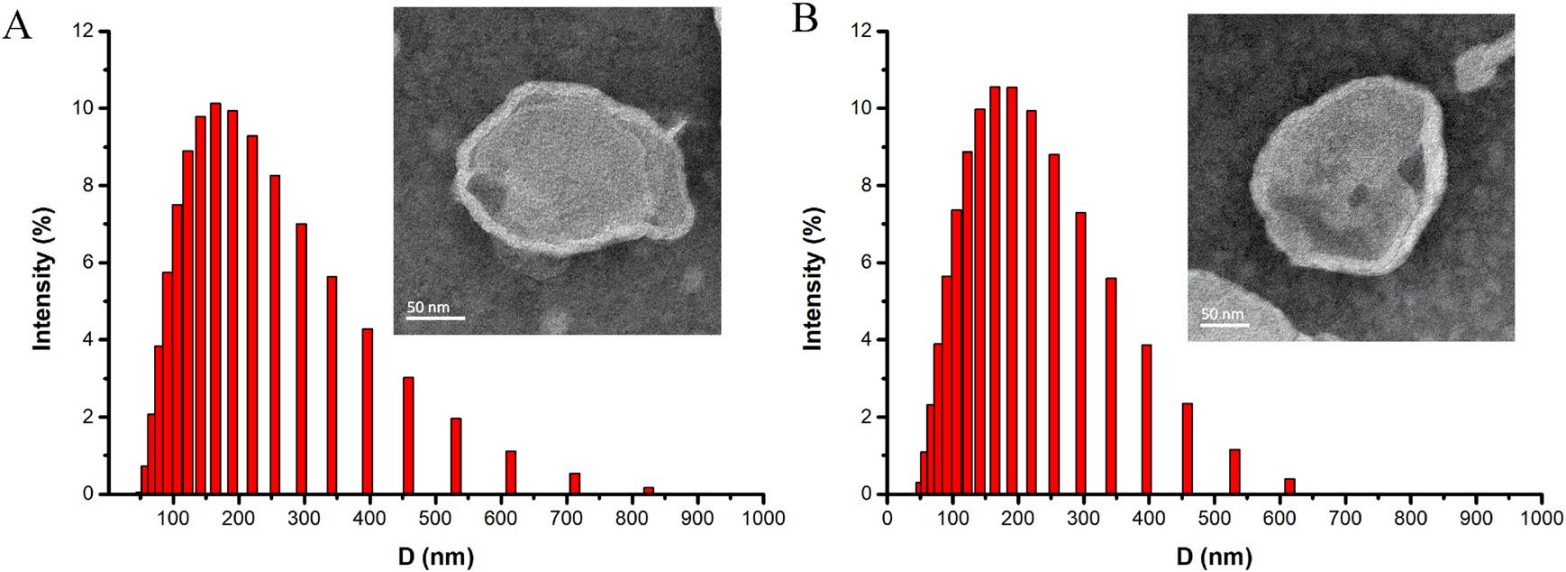
Size distribution and TEM (inset) images of blank PEGylated liposome (A) and PL-Lip (B), respectively.

Compared to blank PEGylated liposomes, the particle size, and Zeta potential of PL loaded PEGylated liposome showed no statistically significant difference (shown in Table 1). The average particle size of PL-Lip was 179.29 ± 4.92 nm, with an acceptable polydispersity index of 0.23 ± 0.037. Moreover, the Zeta potential of PL-Lip was −28.64 ± 3.29 mV, thus showing that the PEGylated liposome possesses desirable physical stability. The DLS result of PL-Lip is shown in Figure 1(A). Furthermore, the TEM micrograph (Figure 1(B)) showed that the PEGylated liposomes droplets were almost spherical unilamelar vesicles in shape. Nonetheless, the encapsulation rate (EL%) of PL-Lip being 91.43 ± 1.57%.

**Table 1.** Physicochemical characterization of blank PEGylated Liposome (B-Lip) and PL-Lip (mean values ± standard deviation, *n* = 3): measurement of mean diameter (d), Zeta potential (z) and polydispersity index (PDI).

Samples	Z[mV]	D[nm]	PDI
B-Lip	−35.83 ± 2.17	180.13 ± 2.49	0.21 ± 0.048
PL-Lip	−28.64 ± 3.29	179.29 ± 4.82	0.23 ± 0.059

### 3.2. The stability of PL-Lip and PL-SNP

**Figure 2.**
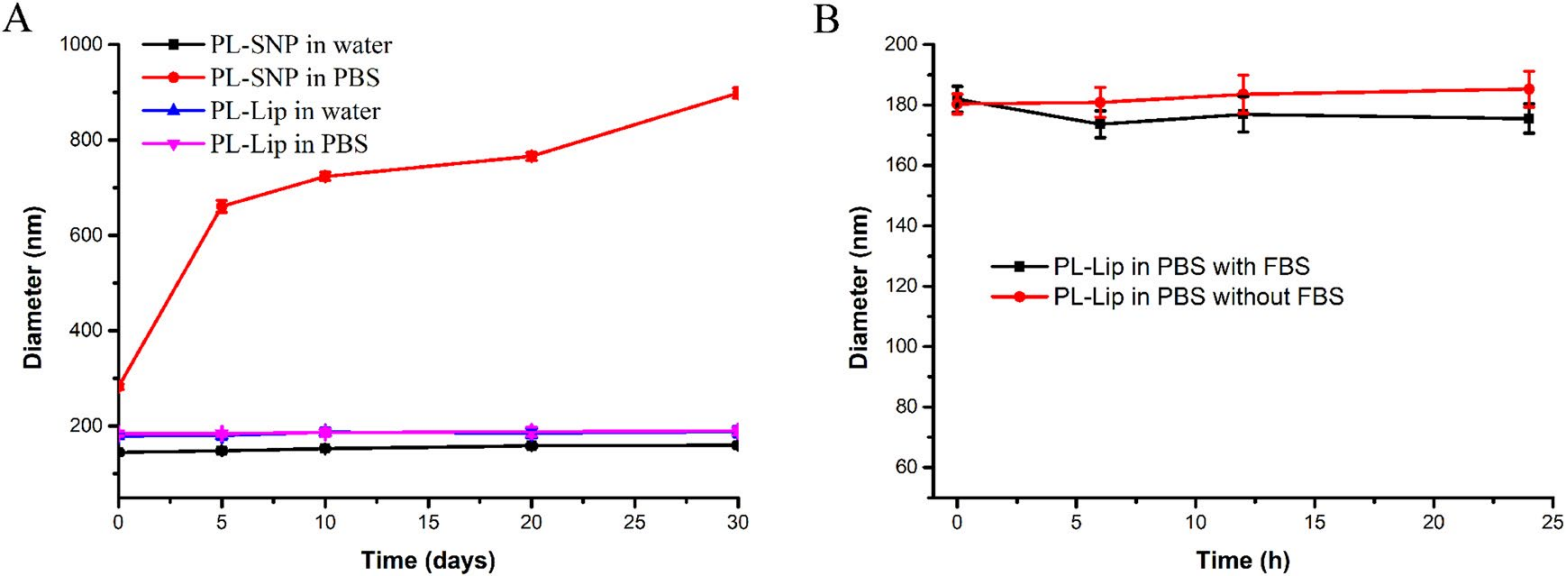
A: The long-term stability of PL-SNP and PL-Lip in water and PBS for 30 days at 4 °C. B: The stability of PL-Lip in PBS with or without 10% FBS at 37 °C for 24 h. The values are expressed as mean ± SD of three determinations.

The long-term stability was investigated in water and PBS (pH = 7.4) at 4 °C. From the results (Figure 2), PL-Lip showed good stability in both water and PBS in a month. However, PL-SNP gradually became larger when the prodrug NPs were transferred into PBS (pH = 7.4) due to the disruption of equilibrium forces between prodrug molecule by ions. Besides, the diameter of PL-Lip did not change significantly over 24 h in both PBS (pH = 7.4) solution and in the absence of FBS at 37 °C. This indicated that PL-Lip could maintain its nano-shape in cell culture medium.

### 3.3. *In vitro* release of PL-Lip and PL-SNP

**Figure 3.**
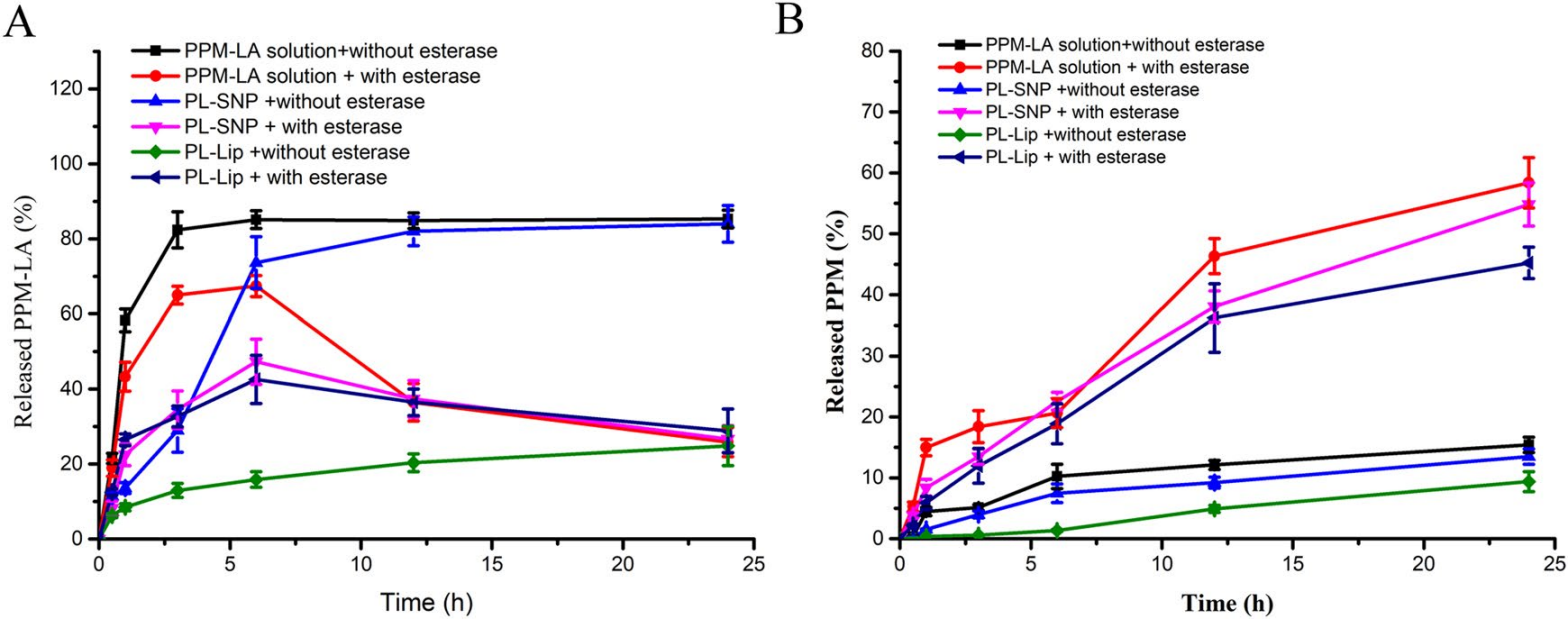
The *in vitro* release profiles of total drugs from PL-SNP and PL-Lip in PBS (7.4) with or without esterase.

The PL prodrug could self-assemble to form nanoparticles (PL-SNP) in water. The PL solution (DMSO) and PL-SNP were used as a comparison sample. The results (Figure 3(A)) showed that PL molecule can quickly pass through the dialysis bag in 4 h. Compared with PL-SNP, PL-Lip showed a slower release rate of PL and PPM due to its better stability in PBS (pH = 7.4) without esterase because eventually the active drug needs to be released to exert pharmacological activity. The PPM and PL release of PL-SNP, PL-Lip in the presence of 10 U esterase were observed over the period of 24 h. The results (Figure 3(B)) showed that the PL released from PL solution, PL-SNP and PL-Lip could be enzymatically hydrolyzed into PPM by esterase. Compared with PL-SNP, PL-Lip showed a similar release rate of PL and PPM in PBS (pH = 7.4) with esterase. Above results indicated PL-Lip could keep stable during the longtime incubation at a low concentration of esterase such as human plasma and slowly release PPM at high low concentration of esterase such as tumor microenvironment (Zhang et al., 2013; Wang et al., 2021).

### 3.4. *In vitro* cytotoxicity

**Figure 4.**
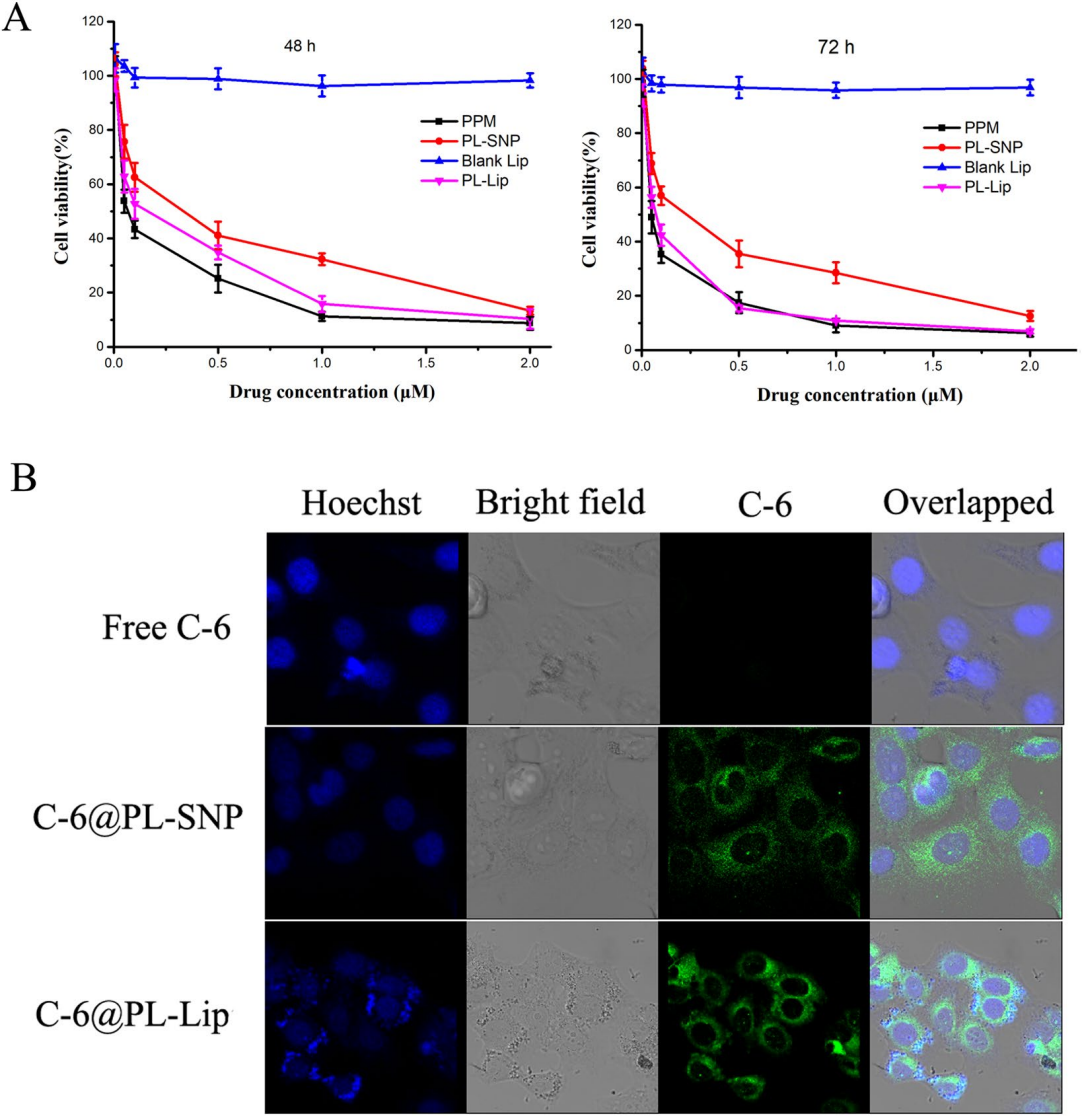
(A) *In vitro* cytotoxic activities of PPM, PL-SNP and PL-Lip against HepG2 human cancer cells. (B) Confocal laser scanning microscopy (CLSM) images of HepG2 cells incubated with free coumarin-6, PL-SNP, or PL-Lip for 3 h.

The *in vitro* cytotoxicity of PPM, blank Lips and PL-SNP or PL-Lip were evaluated on HepG2 using MTT assay. Cell survival results and IC_50_ values are shown in Figure 4(A) and Table 2, respectively. The blank Lips induced almost no cytotoxicity against HepG2 for 48 h or 72 h from 0.005-2 μM. Notably, PPM exhibited highest cytotoxicity to HepG2 cells at 48 and 72 h (IC_50_=0.159 μM/0.113 μM) compared with PL-SNP (IC_50_=0.281 μM/0.223 μM) and PL-Lip (IC_50_=0.218 μM/0.125 μM). While, PL-Lip showed higher cytotoxicity against HepG2 than PL-SNP for both 48 and 72 h. With increasing concentration and time, both PL-SNP and PL-Lip became increasingly cytotoxic. This may be ascribable to a two-step process of PPM released from PL-SNP and PL-Lip occurred, where initially, PL prodrug is slowly released from PL-SNP and PL-Lip, wherein it is slowly converted to PPM with the help of related enzymes in HepG2 cells (Zhang et al., 2018). As can be seen in Figure S1, the IC_50_ on L02 cells for 72 h of PL-SNP (IC_50_=0.28 μM) or PL-Lip (IC_50_=0.24 μM) was significantly higher than that of PPM (IC_50_=0.17 μM) due to the slow release of PPM from PL-SNP or PL-Lip. Altogether, the aforementioned results showed that PL-Lip could decrease cytotoxicity to normal liver cells (L02) and retain the cytotoxicity to tumor cells (HepG2 cells).

**Table 2.** IC_50_ values of PL-SNP and PL-Lip on HepG2 cells in 48 h or 72 h.

Compound	48 h	72 h
	IC_50_ (μM)	IC_50_ (μM)
Blank Lips	>20	>20
PPM	0.159	0.113
PL-SNP	0.281	0.223
PL-Lip	0.218	0.125

NT: not test.

### 3.5. Cell uptake

The C-6 was loaded in prodrug nanoparticles (C-6@PL-SNP) and pegylated liposome (C-6@PL-Lip), while the fluorescence intensity was observed with confocal microscopy to study the cellular uptake of HepG2 cells after 3 h of incubation. The cell uptake result (Figure 4(B)) showed that the cellular fluorescence intensity of C-6@PL-SNP or C-6@PL-Lip in HepG2 was considerably stronger than that of the free C-6 after 3 h. The results indicated that both prodrug nanoparticle and pegylated liposome could improve the internalization of free drug. What is more, C-6@PL-Lip exhibited stronger fluorescence intensity compared with the C-6@PL-SNP. This result may be due to the following factors: 1) C-6@PL-Lip shows better stability than PL-SNP. Instability leads to aggregation of PL-SNP; 2) liposome has stronger cell membrane fusion ability with tumor cell than hydrophobic nanoparticles (Shen et al., 2018).

### 3.6. The pharmacokinetic analysis in rats

**Figure 5.**
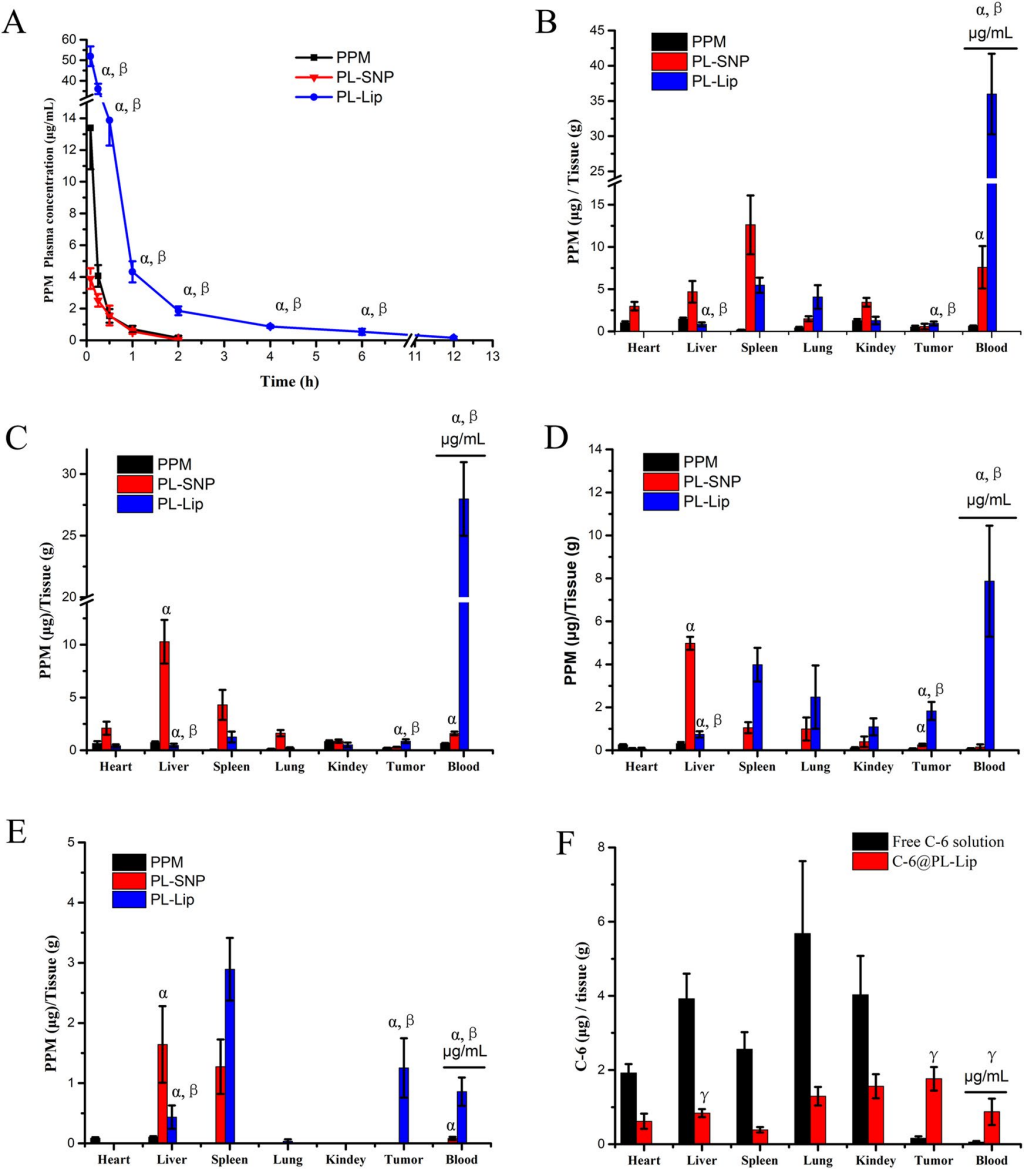
(A) *In vivo* plasma concentration-time profiles of PPM, PL-SNP and PL-Lip after a single intravenous injection in SD rats. (B–E) The PPM content in blood and Tissue of PPM, PL-SNP and PL-Lip after intravenous administration in mice. B: 0.5 h; C: 1 h; D: 2 h; E: 4 h. (F) The C-6 content in blood and tissue after intravenous injection of free C-6 solution and PL-Lip injection in H_22_-bearing mice at 24 h (the data are expressed as the mean ± SD; α represents *p* < .05 compared to PPM group. β represents *p* < .05 compared to PL-SNP group. λ represents *p* < .05 compared to free C-6 solution group, *n* = 5).

In order to investigate the pharmacokinetics parameters of PPM, PL-SNP, and PL-Lip, the plasma concentration-time profiles of the released PPM were evaluated in SD rats after intravenous administration (equivalent to a dose of 4 mg/kg), as shown in Figure 5(A). The data curves were more in line with the non-compartmental pharmacokinetic model. The main pharmacokinetics parameters, with our previous PL-SNP and PPM as comparisons, are presented in Table 3. The values of mean residence time (MRT) and t_1/2_ in PL-Lip animals were enhanced to 2.63 h and 1.55 h, respectively, in comparison to those of the PL-SNP treated animals, which were 0.29 h and 0.45 h, respectively. The above results showed that PL-Lip could significantly improve their retention time in plasma *in vivo*, mainly because the peg-induced surface of liposome could avoid the recognition and reduce the clearance from mononuclear macrophage system (Zahednezhad et al., 2019). However, the *C*_max_ of PL-Lip increased significantly than that of PPM. Notwithstanding, this formulation needs tissue distribution and toxicity experiment *in vivo* to evaluate the safety.

**Table 3.** Pharmacokinetic parameters of PL-SNP and PL-Lip after intravenous administration in rats (mean ± SD).

Parameters	Free drug	PL-SNP	PL-Lip
*C*_max_ (μg·mL^-1^)	13.39 ± 2.60	3.90 ± 0.65	51.98 ± 4.78^b^
*T*_max_ (h)	0.083	0.083	0.083
*t*_1/2_ (h)	0.333 ± 0.05	0.292 ± 0.06	1.547 ± 0.53^b^
MRT (h)	0.332 ± 0.08	0.445 ± 0.12^a^	2.632 ± 0.41^b^
AUC_0-12h_ (h·μg·mL^-1^)	3.712 ± 0.42	2.05 ± 0.37	27.58 ± 3.93^b^

a *p* < .05, compared with free PPM.

b *p* < .01, compared with free PPM.

### 3.7. Biodistribution study in H_22_ tumor-bearing mice model

The tumor targeting was investigated by measuring the tissue drug levels in mice bearing H_22_ tumor with intravenous injection at 0.5, 1, 2, and 4 h points. As shown in Figure 5(B–E), the drug content of PL-SNP was higher in normal and tumor tissues than in free PPM due to the rapid metabolism of bulk drugs. However, PL-SNP was mainly distributed in liver, spleen, and lung, especially liver with abundant mononuclear macrophages. which is consistent with the previous conclusion that nanoparticles were quickly swallowed by mononuclear macrophages (Guo et al., 2020). This also led to a rapid drop of the drug content in blood. In contrast, PL-Lip had higher drug levels in blood and lower drug levels in normal tissue compared to PL-SNP. This demonstrates that PL-Lip reduces drug elimination from the RES and prolongs the system circulation. Therefore, PL-Lip could significantly improve drug distribution in tumor tissues due to EPR effect (Sun et al., 2018; Wang et al., 2020).

To demonstrate the EPR effect of PL-Lip, the C-6 content in tumor of C-6@PL-Lip was measured by HPLC-fluorescent detection at 24 h. As shown in Figure 5(F), the C-6 contents in the heart and liver of C-6@PL-Lip was significantly declined and the C-6 content in tumor was significantly increased compared to the free C-6 solution. What is more, the C-6 content in tumor of C-6@PL-Lip was higher than that of other normal tissue and blood. This indicated that the PL-Lip has certain passive tumor targeting. On the other hand, PL-Lip could improve the low encapsulation rate of PPM drugs and simultaneously co-load other drugs with synergistic such as paclitaxel and doxorubicin (Liu et al., 2016).

## 4. Conclusion

The small molecule prodrug of PL was successfully integrated into PEGylated liposome. The PEGylated liposome achieves high drug loading and colloidal stability. What is more, PL-Lip showed esterase sensitive release of PPM from PL. Furthermore, PL-Lip showed enhanced cytotoxicity and cellular uptake than that of self-assembling nanoprodrug. In addition, PL-Lip could improve the long circulation time and tumor distribution but reduce the normal distribution, especially heart and liver. Therefore, our results suggest that this strategy provide an alternative and effective drug delivery systems for CGs with lower toxicity and higher tumor targeting.

## Supplementary Material

Supplemental Material

Supplemental Material
