# Supplementary figures and images for: Improving cellular uptake and bioavailability of periplocymarin-linoleic acid prodrug by combining PEGylated liposome
Source: Drug Deliv. 2022 Jul 31;29(1):2491–7. doi: 10.1080/10717544.2022.2104406 (PMC9344961; doi:10.1080/10717544.2022.2104406)

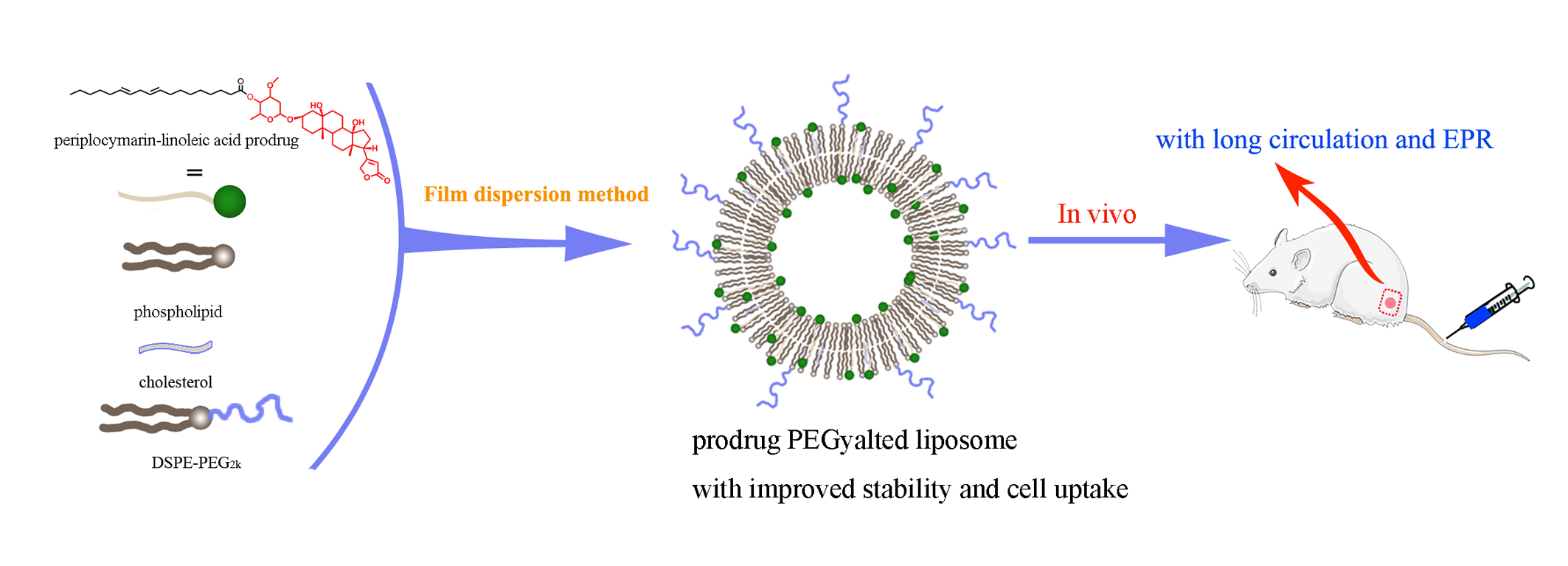

Supplement: Supplemental Material [file IDRD_A_2104406_SM3854.tif]
