# Supplementary material for: Improving cellular uptake and bioavailability of periplocymarin-linoleic acid prodrug by combining PEGylated liposome
Source: Drug Deliv. 2022 Jul 31;29(1):2491–7. doi: 10.1080/10717544.2022.2104406 (PMC9344961; doi:10.1080/10717544.2022.2104406)
Supplement: Supplemental Material [file IDRD_A_2104406_SM3815.docx]

**Supplement**

Improving [cellular](javascript:;) [uptake](javascript:;) and bioavailability of periplocymarin-linoleic acid prodrug by combining PEGylated liposome

Huiyun Zhang ^a,^ *, Shunru Wei^a^, Yu zhang^a^, Anran Pan^a^, Michael Adu-Frimpong^b^, Congyong Sun^c^ and Gang Qi ^a,^*

^a^ School of chemistry and chemical engineering, Yancheng Institute of Technology, Jiangsu, Yancheng 224003, China;

^b.^ Department of Biochemistry and Biotechnology, Kwame Nkrumah University of Science and Technology, Kumasi, Ghana

^c.^ Department of Central Laboratory, The Affiliated Huai’an No.1 People’s Hospital, Nanjing Medical University, 223300 Huai’an, China.

*Corresponding Author’s: Huiyun Zhang, E-mail: [zhanghuiyun1111@](mailto:zhanghuiyun1111@)163.com; Gang Qi, E-mail: [qigang@ycit.cn](mailto:qigang@ycit.cn)

**Methods**

1.The analytical methods of PPM and PPM-LA in vitro and vivo

The concentration of PPM-LA in vitro was measured with an Shimadzu LC-20A HPLC system (Japan) under the following conditions: :C18 column (Spherisorb ODS, 5 μm, 150×4.6 mm, Waters), mobile phase-100% acetonitrile; detection wavelength-220 nm; flow rate-1.0 ml/min; injection volume-20 μL. Based on the preparation of a series of PPM-LA standard solutions with different concentration and the HPLC analysis, standard curve for quantifying PPM-LA was established as follows: A=18236C+384.24, r=0.9998. where A refers to the peak area and C denotes PPM-LA concentration. The linear rang was from 0.1μg⋅mL^-1^ to 100 μg⋅mL^-1^. The concentration of PPM in vitro and in vivo was measured by HPLC as similar as the condition of PPM-LA. The mobile phase consisted of acetonitrile and water (38:62, v/v, containing 0.05% TFA). Standard curve in vitro for quantifying PPM was established as follows: A=32538C-394.6, r=0.9998. where A refers to the peak area and C denotes PPM concentration. The linear rang was from 0.1μg⋅mL^-1^ to 100 μg⋅mL^-1^. Standard curve in vivo for quantifying PPM was A=0.9958C-0.0299: where A refers to the peak area ratio of PPM and bufalin (internal standard) and C denotes PPM concentration. The linear rang was from 0.05μg⋅mL^-1^ to 10 μg⋅mL^-1^.

**The file includes**

1. **Figure S1**





Figure S1 *In vitro* cytotoxic activities of PPM, PL-SNP and PL-Lip against L02 human normal liver cells for 72 h
